# Spectral CT-derived quantitative imaging parameters combined with inflammatory markers for exploratory preoperative grading of bladder cancer

**DOI:** 10.3389/fonc.2026.1892226

**Published:** 2026-08-12

**Authors:** Shuang Qin, Yi Hong, Jie Cheng, Qi Lv

**Affiliations:** 1 Department of Medical Imaging, Shanghai Xuhui Central Hospital/Xuhui Hospital, Fudan University, Shanghai, China; 2 Department of Medical Imaging, Tongji Hospital Affiliated to Tongji University, Shanghai, China; 3 Department of Urology, Shanghai Xuhui Central Hospital/Xuhui Hospital, Fudan University, Shanghai, China

**Keywords:** bladder cancer, gemstone spectral imaging CT, inflammatory markers, iodine concentration, spectral curve slope

## Abstract

**Background:**

Pathological grade and muscular invasion determine bladder cancer treatment and prognosis. Conventional CT lacks quantitative tumor characterization, while gemstone spectral imaging CT (GSI-CT) provides perfusion-related quantitative metrics; systemic inflammatory markers also correlate with tumor aggressiveness.

**Objective:**

To explore whether combined GSI-CT parameters and inflammatory markers improve preoperative bladder cancer grading, and analyze their association with muscular invasion.

**Methods:**

This single-center retrospective study enrolled 61 pathologically confirmed bladder cancer patients (26 low-grade, 35 high-grade) with preoperative GSI-CT. Venous-phase iodine concentration (IC), spectral slope λHU, effective atomic number (Zeff) and four inflammatory indices were measured. Between-group comparisons, logistic regression and DeLong ROC comparison were performed using SPSS 25.0 and R 4.3.3 (α = 0.05).

**Results:**

Venous-phase IC, λHU and Zeff were significantly elevated in high-grade lesions, while lymphocyte-to-monocyte ratio (LMR) was markedly lower (all *P* < 0.05). The λHU + LMR combined model yielded an AUC of 0.877 versus LMR alone (AUC = 0.835), with no statistically significant diagnostic improvement (*P* = 0.061) by DeLong test. After adjusting for tumor diameter, both λHU and LMR remained independent grading predictors. A *post-hoc* inflammation-free subgroup analysis suggested λHU may partially offset the confounding effect of systemic inflammation on LMR performance, though this result is exploratory and requires further validation.

**Conclusions:**

Combined GSI-CT and LMR jointly reflect tumor angiogenesis and host immunity, yet the dual-parameter model offered no statistically meaningful incremental grading value over LMR in this NMIBC-dominated single-center cohort. The auxiliary value of λHU needs large multi-center prospective verification.

## 1 Introduction

Bladder cancer is one of the most common genitourinary malignancies (1), and pathological grading is crucial for guiding clinical treatment (2–4). High-grade bladder cancer carries a high risk of recurrence and progression to muscle-invasive bladder cancer (MIBC), with a 5-year overall survival rate below 50% (5). Current diagnosis relies on a multimodal approach including imaging, cystoscopy, biopsy and urine cytology (6). CT, especially CT urography recommended by the European Association of Urology as the first-line imaging for hematuria patients, is a core tool for bladder cancer evaluation (4, 7).

However, traditional CT relies on morphological features and lacks quantitative assessment capacity, leading to suboptimal accuracy in tumor grade assessment and possible staging underestimation (8). This highlights the need for improved preoperative grading methods to implement personalized treatment. Gemstone Spectral Imaging CT (GSI-CT), an emerging modality, retains high spatial and temporal resolution while adding energy-resolved quantitative imaging. Its key advantages include monochromatic imaging for artifact reduction, material decomposition for tissue quantification, and spectral curve analysis for tissue characterization, enabling more precise tissue property assessment (9, 10).

In bladder cancer research, GSI-CT has shown potential in preoperative evaluation. Li et al. verified that arterial enhancement fraction helps grade bladder tumors (11); Yang et al. found that arterial-phase iodine (water) concentration and normalized values can differentiate bladder urothelial carcinoma grades (12); Yu et al. reported that spectral imaging improves preoperative staging accuracy via optimal monochromatic images (13); other studies confirmed dual-energy CT can identify muscle invasion using quantitative and texture features (14).

Additionally, accumulating evidence indicates that systemic inflammation is linked to tumor progression and prognosis. Inflammation-based blood biomarkers [e.g., neutrophil-to-lymphocyte ratio (NLR), C-reactive protein-to-albumin ratio (CAR), lymphocyte-to-monocyte ratio (LMR), and platelet-to-lymphocyte ratio (PLR)] reflect immune status, correlate with disease outcomes, and are cost-effective to obtain, showing potential as supplementary prognostic tools (15, 16). Although these inflammatory markers are more commonly associated with prognosis, there is a biological rationale for their potential relationship with histologic grade (17). Higher-grade tumors often exhibit more aggressive behavior and stronger tumor-associated inflammatory responses, which may be reflected in peripheral blood inflammatory indices (18). Therefore, these markers were included as complementary indicators for preoperative tumor grading.

Notably, there is a lack of comprehensive research on combining GSI-CT parameters with preoperative inflammatory markers for bladder cancer grade assessment. Thus, this study aims to assess whether integrating GSI-CT-derived quantitative parameters with systemic inflammatory markers can improve the non-invasive prediction of bladder cancer histological grade.

## 2 Materials and methods

### 2.1 General information

This single-center observational study was based on a prospectively collected clinical database, and a retrospective analysis was subsequently performed. Consecutive patients with clinically suspected bladder cancer who underwent preoperative GSI-CT and subsequent surgical treatment at the Department of Urology between August 2023 and August 2025 were enrolled. All enrolled patients were therapy-naïve; none received preoperative radiotherapy, chemotherapy, immunotherapy, or intravesical instillation treatment before GSI-CT scanning and surgery, to eliminate confounding effects of anti-tumor treatment on imaging parameters and peripheral inflammatory markers. All peripheral blood samples for inflammatory marker detection were collected within 3 days before preoperative GSI-CT scanning. The interval between GSI-CT examination and subsequent transurethral resection or radical cystectomy surgery was controlled within 14 days for all enrolled patients.

Histopathological diagnosis and tumor grading were established based on surgical specimens obtained by transurethral resection of bladder tumor (TURBT) or radical cystectomy, and were evaluated by experienced pathologists in accordance with the 2016 WHO classification (19).

The study was approved by the Institutional Review Board (Ethics Approval No. 2021-119, No. K-2025-074) and conducted in accordance with the Declaration of Helsinki. Written informed consent was obtained from all patients.

Patients were included if they had: (1) pathologically confirmed bladder urothelial carcinoma with complete pathological results; (2) complete and diagnostically adequate GSI-CT data; (3) available clinical laboratory data of systemic inflammatory markers; and (4) no prior anti-tumor treatment.

Patients were excluded if they had: (1) non-urothelial or variant histology; (2) other concurrent malignancies; (3) recurrent bladder cancer; (4) preoperative anti-tumor therapy; or (5) incomplete or non-diagnostic CT data (e.g., due to motion artifacts, inadequate bladder filling, or technical issues), which were excluded to ensure reliable quantitative analysis.

Collected data included demographic characteristics, pathological findings, GSI-CT quantitative parameters, and laboratory indicators.Notably, predefined prospective exclusion criteria for patients with inflammatory confounders were not established during study enrollment. To retrospectively quantify the interference of inflammatory status on LMR values, we conducted a *post-hoc*, hypothesis-generating sensitivity analysis (this subgroup stratification was not pre-specified at study design stage). We retrospectively reviewed complete electronic medical records to identify and exclude 11 patients with confirmed active urinary tract infection, chronic systemic inflammatory disorders, or systemic corticosteroid use within two weeks prior to peripheral blood collection, forming an inflammation-free restricted cohort (n = 50) for supplementary exploratory evaluation.

For the *post-hoc* inflammation-free subgroup screening, we comprehensively reviewed complete electronic medical records, laboratory test reports and clinical medication records of all 61 patients according to uniform identification criteria: (1) Urinary tract infection: Defined as positive urine routine leukocyte/erythrocyte counts, or positive urine bacterial culture with corresponding lower urinary tract symptoms recorded in medical history; (2) Chronic systemic inflammatory disorders: Confirmed clinical diagnosis of rheumatoid arthritis, inflammatory bowel disease, chronic nephritis or other chronic autoimmune inflammatory diseases documented in past medical history; (3) Corticosteroid use: Any oral or intravenous glucocorticoid administration recorded within 14 days prior to peripheral blood collection.

Patients meeting any one of the above three criteria were excluded from the inflammation-free restricted cohort, leaving 50 participants for supplementary exploratory analysis. All screening judgements were independently verified by two clinicians to avoid subjective misclassification.

### 2.2 Research methods

#### 2.2.1 CT scanning protocol

In this study, all subjects underwent imaging examinations using the GE Discovery CT 750 HD scanning system, including conventional plain scan and spectral three-phase dynamic enhanced scan. During the examination, the subjects maintained a supine position, and the scanning direction was from head to toe, with the scanning range extending from the top of the diaphragm to the level of the pubic symphysis. The specific parameter settings were as follows: (1) The conventional plain scan adopted a fixed voltage of 120 kVp and intelligent mA adjustment technology. (2) The spectral scan enabled the gemstone spectral imaging mode, with technical parameters including instantaneous switching of dual energy (80/140 kVp), 0.625 mm collimation width, 600 mA constant current, 0.6 seconds/rotation scanning speed, and 0.983 pitch ratio. (3) All image data were reconstructed three-dimensionally with a slice thickness of 1.25 mm and equal spacing.

The intravenously injected contrast agent was iohexol (300 mg I/mL) at an injection flow rate of 3.0 mL/s, and the total dose was calculated at 1.5 mL/kg body weight.

Timing for Each Phase: (1) Arterial phase: 30 seconds post-contrast injection. (2) Venous phase: 120 seconds post-contrast injection.

The fixed arterial delay and venous delay represent standardized timing protocols widely adopted in bladder gemstone spectral CT examinations as previously reported, ensuring adequate contrast opacification of bladder lesions and adjacent soft tissues for quantitative parameter measurement (20, 21).

Notably, this study adopted a uniform fixed arterial delay without automated bolus tracking, which constitutes an important technical limitation for our elderly cohort (mean age = 70.03 years). Patients exhibited highly variable cardiac output and systemic circulation speeds; the fixed 30-second delay often failed to capture peak arterial enhancement of tumor tissue, introducing measurement variability that likely attenuated arterial-phase discriminatory signals between high- and low-grade bladder Tumors.

#### 2.2.2 Image processing and analysis

In the quantitative analysis of GSI-CT images, two radiologists with more than 5 years of experience independently reviewed the images. First, the slice showing the maximum tumor diameter was selected on axial images. A circular region of interest (ROI) was manually delineated within the uniformly enhanced solid component of the lesion, following these principles: (1) covering ≥2/3 of the maximum cross-sectional area; (2) avoiding non-enhanced regions (e.g., cystic changes, necrosis, or hemorrhage); and (3) ensuring consistent ROI positioning across all imaging phases.

Interobserver agreement for quantitative measurements was assessed using the intraclass correlation coefficient (ICC), with ICC > 0.75 indicating good agreement. In this study, all parameters demonstrated good interobserver agreement, and the mean values of the two observers were used for subsequent analysis.

The quantitative parameters analyzed included: (1) monochromatic CT values at 40–70 keV (10 keV intervals) in arterial and venous phases; (2) iodine concentration (IC) within the lesion, as well as iodine concentration in the internal iliac artery at the same level (IC_artery); and (3) effective atomic number (Zeff).

Derived parameters were calculated as follows: (1) slope of the spectral curve (λHU) = (CT_40keV − CT_70keV)/30; and (2) normalized iodine concentration (NIC) = IC/IC_artery.

#### 2.2.3 Statistical methods

Statistical analysis was performed using SPSS version 25.0 (IBM Corp., Armonk, NY, USA) and R version 4.3.3 (R Foundation for Statistical Computing, Vienna, Austria). Interobserver agreement for quantitative GSI-CT measurements was assessed using the intraclass correlation coefficient (ICC). An ICC > 0.75 indicated good agreement, 0.40–0.75 indicated moderate agreement, and < 0.40 indicated poor agreement. For parameters with ICC > 0.75, the mean values of the two observers were used for subsequent analysis. The Shapiro–Wilk test was used to assess data normality. Normally distributed variables were expressed as mean ± standard deviation ( $\bar{x}$ ± s) and compared using the independent-samples t-test. Non-normally distributed variables were expressed as median (interquartile range) and compared using the Mann–Whitney U test. A p value < 0.05 was considered statistically significant. Receiver operating characteristic (ROC) curve analysis was performed to evaluate the diagnostic performance of each parameter, and the area under the curve (AUC) with 95% confidence intervals was calculated. To construct a combined diagnostic model incorporating GSI-CT parameters and inflammatory markers, multivariate logistic regression analysis was performed. Considering the relatively small sample size and potential risk of small-sample bias, Firth bias-reduced logistic regression was additionally performed as a sensitivity analysis, and the results were generally consistent with those of the standard logistic regression model. The results of the Firth model were compared with those of the standard logistic regression model to assess robustness. Internal validation of the model was conducted using 5-fold cross-validation, and the mean cross-validated AUC was calculated to assess model stability. Model calibration was evaluated using the Hosmer–Lemeshow goodness-of-fit test and the Brier score. The predicted probabilities derived from the final logistic regression model were used to construct the ROC curve and to calculate the AUC, sensitivity, and specificity.

Multiple inflammatory markers (LMR, NLR, CAR, PLR) and spectral CT parameters (venous/arterial IC, NIC, λHU, Zeff) were compared between high- and low-grade groups. To control type I error inflation induced by multiple comparisons, the false discovery rate (FDR) Benjamini–Hochberg correction was applied for all between-group comparisons of imaging and inflammatory indicators. The original significance threshold α = 0.05 was retained for adjusted P values; unadjusted P values were also reported alongside corrected P values for transparency.

The bootstrap procedure was used to evaluate the internal stability of model performance by estimating the distribution of AUC values across 1000 bootstrap resamples.

Sensitivity analysis. To evaluate the potential influence of patients who underwent radical cystectomy, an additional sensitivity analysis was performed after excluding these nine patients. The λHU + LMR logistic regression model was refitted in the remaining TURBT cohort, and the corresponding ROC analysis was repeated.

Tumor diameter was included as a continuous adjusting covariate to verify whether the predictive value of λHU and LMR for pathological grade is independent of lesion volume. Tumor multifocality was not included in the primary model due to the small sample size and low event rate of multifocal disease, and its potential confounding effect is acknowledged in the Limitations section.

## 3 Results

### 3.1 General information

**Figure 1:**
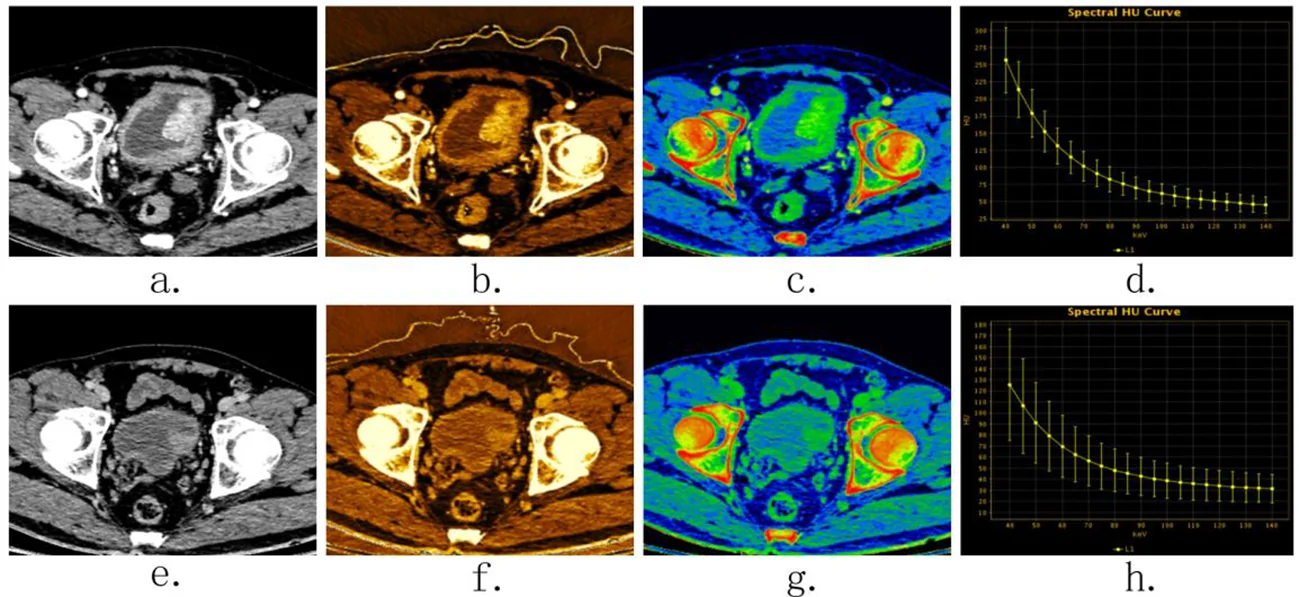
GSI-CT imaging and spectral curve: **(A–D)** Male, 71 years old, high-grade urothelial carcinoma of the bladder. Venous phase: **(A)** 40keV image, the monochromatic CT value of the lesion is 254.15 HU; **(B)** Iodine map, IC value is 2.744 mg/mL; **(C)** Effective atomic number map, Zeff value is 9.145; **(D)** Spectral curve graph, λHU is 5.16. **(E–H)** Male, 61 years old, low-grade urothelial carcinoma of the bladder. Venous phase: **(E)** 40keV image, the monochromatic CT value of the lesion is 122.86 HU; **(F)** Iodine map, IC value is 1.1915 mg/mL; **(G)** Effective atomic number map, Zeff value is 8.33; **(H)** Spectral curve graph, λHU is 2.239.

Among 83 screened patients, 61 were eligible for final analysis. The mean age was 70.03 ± 9.83 years overall. The cohort included 52 male patients (85.2%) and 9 female patients (14.8%). All patients underwent surgical treatment within 30 days after CT examination. According to the 2016 WHO classification, 35 patients (57.4%) had high-grade and 26 (42.6%) had low-grade bladder cancer. Of all high-grade cases, 7 patients were confirmed to have concomitant carcinoma *in situ* (CIS); consistent with WHO diagnostic criteria, CIS lesions were categorized as high-grade disease in this cohort. A total of 19 patients presented multifocal bladder Tumors, while the remaining 42 had solitary lesions. Based on TNM staging, 43 patients (70.5%) had non–muscle-invasive bladder cancer (NMIBC, Ta/T1) and 18 (29.5%) had muscle-invasive bladder cancer (MIBC, ≥T2). Regarding the choice of surgical methods, 85.2% (52/61) of the cases underwent transurethral resection of bladder tumor (TURBT), and the remaining 14.8% (9/61) underwent radical cystectomy. All 61 enrolled patients completed standardized preoperative GSI-CT scanning, and a total of 61 valid CT examinations were included for quantitative image analysis. Detailed clinical characteristics are shown in the table (**Table 1**). The GSI-CT Imaging and Spectral Curve images of two of the patients are shown in the **Figure 1**.

**Table 1:** General information of patients.

Clinicopathological indicators	Group	Number of cases	%
Gender	Male	52	85.2
	Female	9	14.8
Age (mean ± SD)	70.03 ± 9.83		
Tumor muscular invasion status	Muscular invasion	18	29.5
	Non-muscular invasion	43	70.5
urothelium carcinoma	Carcinoma in situ	7	11.5
	Infiltrating carcinoma	54	88.5
Tumor diameter	< 3cm	48	78.7
	≥ 3cm	13	21.3
Muscle invasion status	MIBC	18	29.5
	NMIBC	43	70.5
Histological grade	High-grade	35	57.4
	Low-grade	26	42.6
Surgical method	TURBT	52	85.2
	radical cystectomy	9	14.8

### 3.2 Inter-observer consistency analysis

To assess the repeatability of quantitative measurements, ICCs were calculated for inter-observer assessments between the two radiologists. IC, λHU, Zeff, LMR in high-grade tumors showed excellent interobserver reproducibility, with ICCs of 0.954 (95% CI: 0.879-0.983), 0.983(95% CI: 0.957-0.994), 0.977 (95% CI: 0.941-0.991), and 0.952 (95% CI: 0.872-0.982), respectively. IC, λHU, Zeff, LMR in low-grade tumors showed excellent interobserver reproducibility, with ICCs of 0.979 (95% CI: 0.930-0.994), 0.986 (95% CI: 0.953-0.996), 0.981 (95% CI: 0.953-0.996), and 0.969 (95% CI: 0.971-0.984), respectively.

### 3.3 Differences in GSI-CT parameters and inflammatory markers between high-grade and low-grade groups

Among the 61 patients included in this study, pathological results showed 35 cases in the high-grade group (57.4%) and 26 cases in the low-grade group (42.6%). Regarding inflammatory markers, the LMR was significantly lower in the high-grade group than in the low-grade group (*P* < 0.05). No significant differences were observed in NLR, CAR, or PLR between the two groups (*P* > 0.05). Four systemic inflammatory markers (LMR, NLR, CAR, PLR) were analyzed in parallel, which raises the risk of multiple comparison bias. After Benjamini–Hochberg FDR correction for multiple testing, only LMR remained significantly different between high- and low-grade Tumors (adjusted *P* < 0.05), while NLR, CAR and PLR still showed no intergroup difference (adjusted *P* > 0.05). This consistent finding eliminates false-positive risk arising from four parallel inflammatory marker comparisons.

GSI-CT analysis demonstrated that the overall trend of the spectral attenuation curves was similar in both arterial and venous phases between high-grade and low-grade tumors. The most notable differences in CT attenuation were observed in the 40–70 keV energy range.

In the venous phase, the IC was significantly higher in the high-grade group than in the low-grade group (*P* < 0.05), while no significant difference was found in NIC. Additionally, λHU within the 40–70 keV range was significantly greater in the high-grade group compared to the low-grade group (*P* < 0.05). Analysis of Zeff values showed a significantly higher mean Zeff in the high-grade group (8.81 ± 0.22) than in the low-grade group (8.65 ± 0.16) (*P* < 0.05) (**Table 2**).

**Table 2:** Differences in GSI-CT parameters and inflammatory markers between high-grade and low-grade groups.

Spectral parameters	High-grade(n = 35)	Low-grade(n = 26)	*P*	Adjusted *P* (FDR)
IC in the arterial phase (mg/mL)	1.38 (1.27–1.61)	1.47 (1.29–1.59)	0.9360	*P >* 0.05
NIC in the arterial phase	0.16 ± 0.03	0.15 ± 0.04	0.7782	*P >* 0.05
λHU in the arterial phase	2.76 ± 0.77	2.63 ± 0.63	0.4902	*P >* 0.05
IC in the venous phase (mg/mL)	2.11 (2.01–2.38)	1.83 (1.77–1.97)	0.0002	*P* < 0.05
NIC in the venous phase	0.61 ± 0.12	0.57 ± 0.13	0.186	*P >* 0.05
λHU in the venous phase	3.98 (3.79–4.48)	3.43 (3.35–3.71)	0.0001	*P* < 0.05
Zeff	8.81 ± 0.22	8.65 ± 0.16	0.0029	*P* < 0.05
LMR	3.37 ± 0.72	4.41 ± 0.75	<0.0001	*P* < 0.05
NLR	2.23 (1.94–2.68)	2.05 (1.64–2.47)	0.3394	*P >* 0.05
CAR	0.06 (0.02–0.16)	0.04 (0.01–0.08)	0.1606	*P >* 0.05
PLR	52.9 (43.5–60.4)	48.11 (40.49–61.74)	0.7705	*P >* 0.05

However, in the arterial phase, no statistically significant differences were observed between the two groups in IC, NIC, or λHU values (P > 0.05).

To further assess diagnostic performance, ROC curve analysis was performed using venous phase IC, λHU (40–70 keV), Zeff, and LMR values.

### 3.4 ROC curve analysis and diagnostic performance comparison

ROC curve analysis was performed to evaluate the diagnostic performance of individual and combined parameters in distinguishing high-grade from low-grade bladder cancer. The AUC, optimal cutoff value, sensitivity, and specificity were calculated for each parameter.

In the venous phase, IC yielded an AUC of 0.784 (95% CI: 0.661-0.896). Using an optimal threshold of 2.01 mg/mL, IC achieved a specificity of 77.1% and a sensitivity of 76.9%. The λHU within the 40–70 keV range demonstrated superior diagnostic performance, with an AUC of 0.801 (95% CI: 0.682-0.912). At the optimal cutoff of 3.76, λHU provided a sensitivity of 80.0% and a specificity of 76.9%. The Zeff showed an AUC of 0.754 (95% CI: 0.612-0.873), with an optimal threshold of 8.72, a sensitivity of 82.9%, and a specificity of 69.2%. Among the inflammatory markers, the LMR had an AUC of 0.835 (95% CI: 0.727–0.929), with sensitivity (88.6%) and specificity (69.2%) (**Table 3**).

**Table 3:** Diagnostic efficacy of GSI-CT venous phase parameters and inflammatory markers between high-grade and low-grade groups.

Parameter	AUC(95%CI)	Cutoff	Sensitivity(%)	Specificity(%)
IC	0.784(0.661–0.896)	2.01	77.1	76.9
λHU	0.801(0.682–0.912)	3.76	80.0	76.9
Zeff	0.754(0.612– 0.873)	8.72	82.9	69.2
LMR	0.835(0.727–0.929)	4.21	88.6	69.2
combined model (IC +λHU + Zeff + LMR)	0.873(0.781–0.954)	0.58	82.9	76.9
simplified combined model (λHU + LMR)	0.877(0.787–0.955)	0.69	74.3	84.6

For the combined λHU + LMR model, EPV = 17.5 (35 outcome events/2 predictors), meeting the ≥10 threshold. 1000-bootstrap validation: mean AUC = 0.884, 95% CI [0.793, 0.956], SD = 0.042.

Before constructing the multivariate logistic regression model, variance inflation factor (VIF) analysis was performed to quantify multicollinearity among venous-phase IC, λHU, and Zeff. The calculated VIF values for IC, λHU, and Zeff were 7.26, 8.13, and 6.87, respectively. All values exceeded the standard threshold of 5, confirming moderate-to-high multicollinearity among these iodine-dependent spectral parameters, which share the same underlying biological signal of intratumoral iodine accumulation and tumor perfusion.

**Figure 2:**
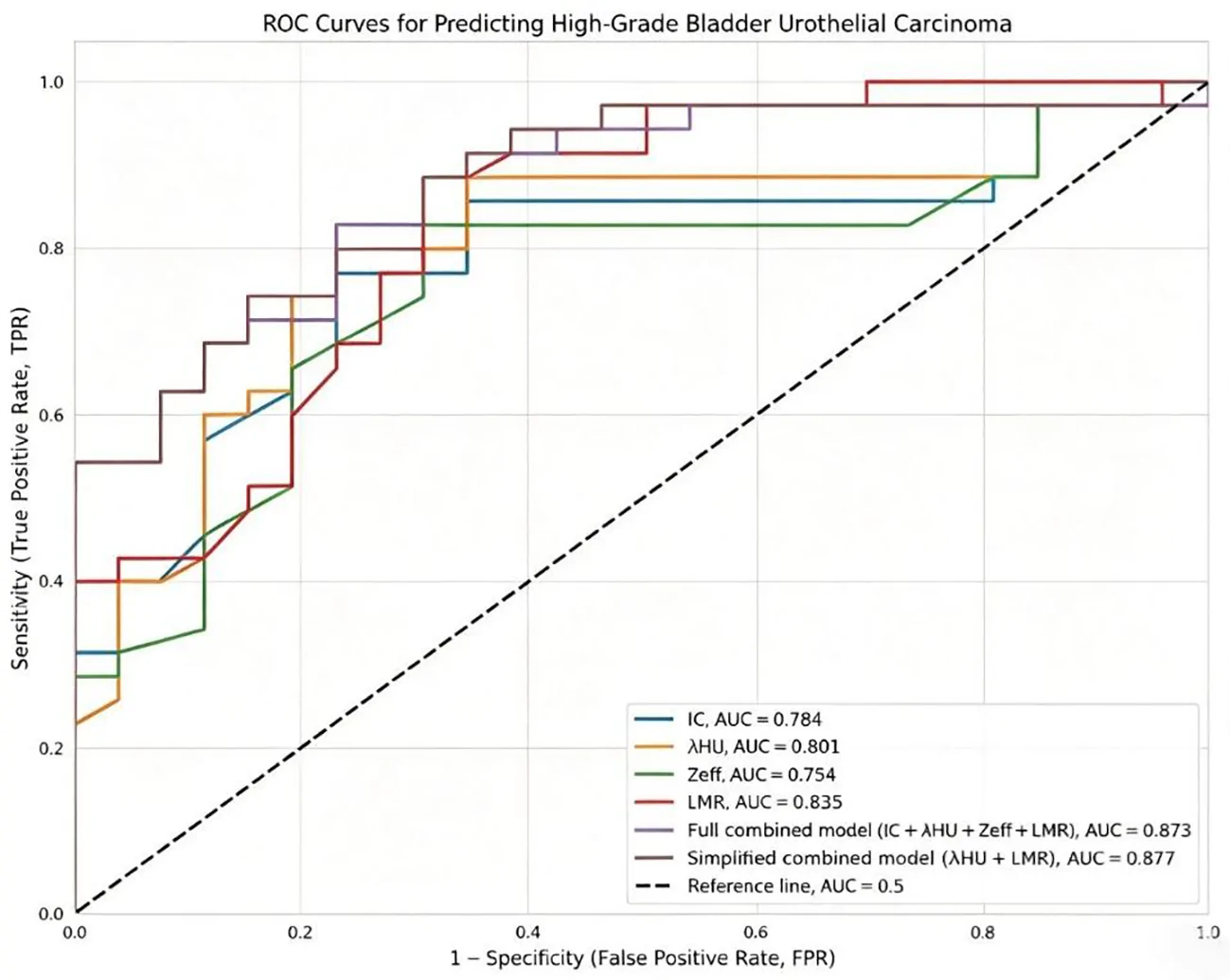
ROC curves of diagnostic efficacy of GSI-CT venous phase parameters and inflammatory markers between high-grade and low-grade groups.

To eliminate statistical bias caused by collinear predictors, two logistic regression models were established for comparison: Model 1 (full model incorporating IC, λHU, Zeff and LMR); Model 2 (simplified model retaining only λHU— the top-performing individual spectral parameter combined with LMR). The full collinear model produced an AUC of 0.873, whereas the simplified λHU + LMR model yielded an AUC of 0.877. The difference in diagnostic efficacy between the two models was negligible. Considering severe multicollinearity among the three spectral metrics, we ultimately adopted Model 2 as the primary predictive model for this study, as it avoids unstable regression coefficient estimates while retaining nearly all predictive power for tumor grade discrimination (**Figure 2**).

#### 3.4.1 Primary analysis in the full cohort

The primary statistical finding of this full-cohort ROC comparison is the absence of statistically significant incremental diagnostic gain when adding venous λHU to standalone LMR. We performed DeLong’s test for paired ROC curves to compare the simplified λHU + LMR combined model (AUC = 0.877) against LMR alone (AUC = 0.835), yielding P = 0.061, which exceeded the pre-specified significance threshold of 0.05. Although the combined model demonstrated a small numerical AUC advantage, this marginal improvement lacked statistical evidence, indicating standalone LMR achieved comparable overall Tumor grading performance without the need for contrast-enhanced spectral CT imaging, associated radiation exposure, extra costs, and time-consuming quantitative post-processing. Supplementary exploratory subgroup analyses detailed below provide only tentative, unvalidated signals and cannot override this main null cohort result.

#### 3.4.2 *Post-hoc* sensitivity analysis in the systemic inflammation-free subgroup

This subgroup analysis is exploratory and cannot be used to justify the primary claim of the combined model’s superiority; it is only intended to generate hypotheses for future validation research.

In the restricted cohort of 50 patients without systemic inflammatory confounders, LMR still remained a significant differentiator of Tumor grade (*P* < 0.001), with an AUC of 0.812, slightly lower than the 0.835 observed in the full dataset. The combined diagnostic model’s AUC marginally declined to 0.859 after exclusion of these confounder-affected individuals. Preliminary observation from this restricted cohort suggests that λHU may partially offset the reduced LMR performance caused by systemic inflammatory interference, but this finding requires independent prospective validation in larger cohorts before any clinical application can be considered.

**Figure 3:**
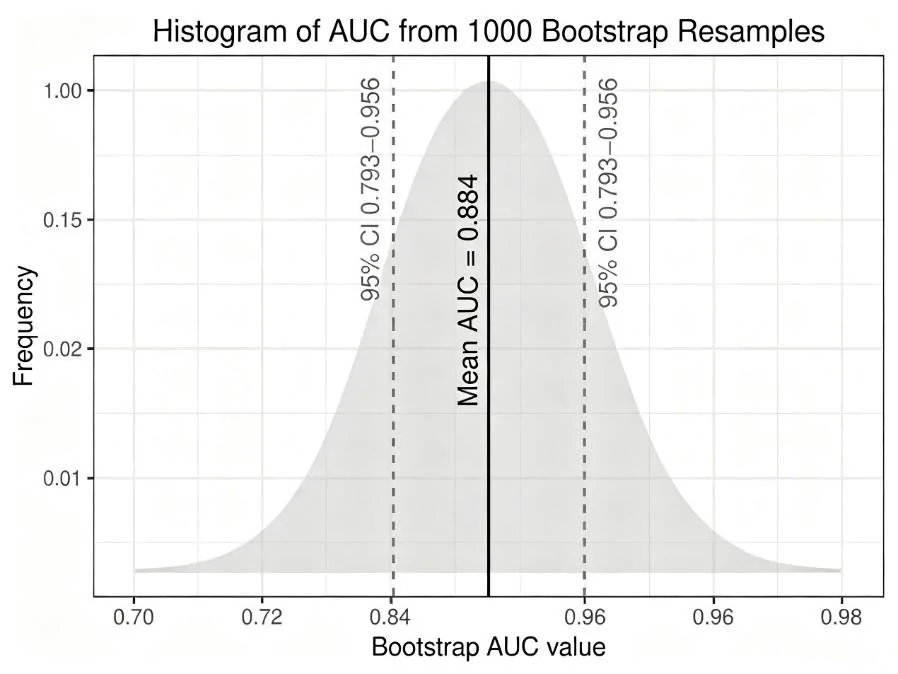
Histogram of AUC values obtained from 1000 stratified bootstrap resamples for the primary simplified combined model (λHU + LMR). The horizontal dashed line represents the mean bootstrap AUC (0.884), and two vertical dotted lines denote the lower and upper bounds of the 95% CI (0.793–0.956). The narrow distribution of AUC values demonstrates stable predictive performance and minimal risk of overfitting.

**Figure 4:**
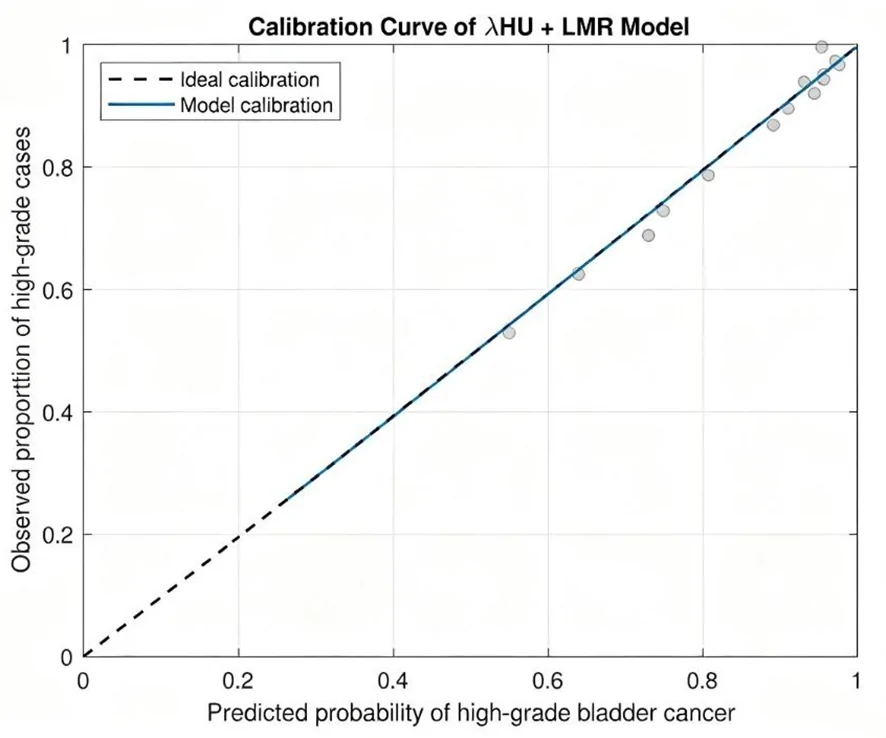
Calibration plot for the primary λHU + LMR logistic regression model. The x-axis shows predicted probability of high-grade bladder cancer, and the y-axis represents the observed proportion of high-grade cases. The solid smooth curve is the model calibration line, and the diagonal dashed ideal reference line indicates perfect prediction consistency. Close overlap between the two lines confirms satisfactory model calibration without systematic prediction bias.

The simplified two-variable model (λHU + LMR) yielded an AUC of 0.877 in the full cohort. To further validate the stability and generalizability of the model, 1000-times stratified bootstrap resampling was performed. The bootstrap validation results are as follows: mean AUC = 0.884, median AUC = 0.887, AUC standard deviation = 0.042, 95% confidence interval for AUC = [0.793, 0.956]. The narrow confidence interval and low standard deviation confirm the excellent stability of the model’s predictive performance, suggesting acceptable internal stability of the model. The model calibration curve demonstrated strong alignment between predicted high-grade probabilities and actual observed outcomes, verifying the reliability of the model’s probability predictions (**Figures 3**, **4**).

### 3.5 Exploratory subgroup analysis by tumor stage

To further explore the performance of the λHU + LMR combined model in different clinical stages, we stratified the full cohort into non-muscle-invasive bladder cancer (NMIBC, Ta/T1) and muscle-invasive bladder cancer (MIBC, ≥T2) subgroups based on muscular invasion status.

#### 3.5.1 NMIBC subgroup (Ta/T1, n = 43)

This subgroup included 25 low-grade tumors and 18 high-grade tumors. Univariate analysis showed that both core parameters were significantly different between low-grade and high-grade groups: λHU (low-grade 3.40 ± 0.55 vs high-grade 4.08 ± 0.85, *P* = 0.0057) and LMR (low-grade 4.45 ± 0.74 vs high-grade 3.70 ± 0.47, *P* = 0.0002). ROC curve analysis showed that standalone LMR achieved an AUC of 0.789, while the λHU + LMR combined model yielded an AUC of 0.864. DeLong’s test showed no statistically significant difference in discriminatory performance between the combined model and standalone LMR (*P* = 0.1538), which was consistent with the result of the full cohort analysis.

#### 3.5.2 MIBC subgroup (≥T2, n = 18)

This subgroup included only 1 low-grade tumor and 17 high-grade tumors, with an extremely imbalanced sample size and insufficient statistical power for formal grading comparison. Therefore, we only present descriptive statistics for this subgroup, and no valid statistical inferences can be drawn regarding the performance of the combined model in the MIBC population.

### 3.6 Sensitivity analysis excluding radical cystectomy patients

To assess whether the inclusion of patients undergoing radical cystectomy influenced the core results of the study, we repeated the primary analysis after excluding these 9 patients, leaving a restricted cohort of 52 patients who only received TURBT.

In the univariate analysis of the restricted TURBT-only cohort, both core parameters remained significantly different between high-grade and low-grade groups: λHU (high-grade 4.05 ± 0.82 vs low-grade 3.40 ± 0.55, *P* = 0.0057) and LMR (high-grade 3.68 ± 0.49 vs low-grade 4.45 ± 0.74, *P* < 0.001).

In the multivariate logistic regression analysis, both λHU and LMR remained independently associated with pathological grade: λHU (OR = 3.27, 95%CI 1.42–7.51, *P* = 0.005) and LMR (OR = 0.41, 95%CI 0.25–0.67, *P* < 0.001).

The ROC curve analysis showed that the λHU + LMR combined model achieved an AUC of 0.872 (95% CI: 0.779–0.951) in the restricted cohort, while standalone LMR yielded an AUC of 0.829 (95% CI: 0.718–0.925). DeLong’s paired ROC comparison produced a P-value of 0.064, which exceeded the 0.05 significance threshold.

Overall, the diagnostic performance and statistical trend of the restricted cohort were highly comparable to those observed in the complete cohort, confirming the robustness of our primary conclusions. Full diagnostic parameters of this sensitivity analysis are summarized in **Supplementary Table 1**.

In the multivariate logistic regression analysis without Tumor diameter adjustment, both λHU (OR = 3.27, 95%CI 1.42–7.51, *P* = 0.005) and LMR (OR = 0.41, 95%CI 0.25–0.67, *P* < 0.001) remained independently associated with pathological grade. After further adjusting for continuous Tumor diameter in the multivariate model, λHU (OR = 2.98, 95%CI 1.31–6.78, *P* = 0.009) and LMR (OR = 0.45, 95%CI 0.28–0.72, *P* = 0.001) still maintained independent predictive significance for high-grade bladder cancer; Tumor diameter itself was also an independent risk factor for high-grade disease (OR = 1.72, 95%CI 1.12–2.63, *P* = 0.013). The diameter-adjusted λHU + LMR combined model yielded an AUC of 0.892, slightly superior to the unadjusted model (AUC = 0.872). All adjusted regression and diagnostic parameters are summarised in **Supplementary Table 2**.

## 4 Discussion

Pathological grading based on TURBT specimens remains the indispensable gold standard for clinical decision-making. This invasive procedure has drawbacks, including patient discomfort, procedural risks, and sampling errors that may underestimate tumor grade or stage (22), highlighting the need for reliable non-invasive preoperative characterization methods (23). The GSI-CT parameters combined with inflammatory markers in this study provide non-invasive, supplementary preoperative information but cannot replace histopathological evaluation.

MRI is currently considered the key imaging modality for evaluating bladder wall muscular invasion and plays a central role in clinical decision-making. However, it may be time-consuming and less well tolerated in elderly or comorbid patients due to claustrophobia or physical limitations (24). Conventional CT, while widely available and rapid, lacks sufficient soft tissue resolution and is limited in assessing tumor biological characteristics and local invasion (25).

GSI-CT, an advanced dual-energy imaging technique, enables multi-parametric tissue characterization through energy-dependent attenuation and material decomposition. It allows quantitative evaluation of parameters such as IC, λHU, and Zeff (26), which may reflect tumor perfusion, vascularity, and composite tissue characteristics associated with tumor biology (27).

In recent years, GSI-CT has shown potential in lesion detection and characterization across various diseases (28). In bladder cancer, previous studies have suggested its value in tumor characterization; however, most investigations remain limited to imaging-based analyses (29). The potential role of combining multi-phase GSI-CT parameters with systemic biomarkers for assessing tumor biological behavior has not been fully explored (24).

### 4.1 GSI-CT quantitative parameters and tumor grade

The core finding of this cohort analysis is that the λHU + LMR combined model did not produce a statistically significant improvement in grading performance compared with LMR alone (AUC 0.877 vs 0.835, *P* = 0.061). Numerically higher AUC of the combined model failed to reach the pre-set statistical threshold, indicating that adding spectral CT metrics could not provide verified additional grading benefit in general bladder cancer patients. Although λHU can reflect lesion-specific perfusion unaffected by systemic inflammation and slightly offset LMR’s performance decline in the inflammation-free exploratory subgroup, this *post-hoc* observation is preliminary and cannot override the primary negative result. Single LMR is a low-cost, radiation-free biomarker with comparable discriminatory power, while routine GSI-CT brings extra radiation and imaging workload without proven incremental clinical value based on current data.

Venous-phase IC, λHU and Zeff were all significantly elevated in high-grade tumors, consistent with enhanced intratumoral angiogenesis and iodine retention in aggressive lesions. Due to strong multicollinearity among IC, λHU and Zeff (VIF > 5), we selected λHU as the representative spectral marker to construct the simplified combined model and avoid unstable regression coefficients. All spectral metrics were measured on 2D axial ROIs without 3D imaging-pathology co-registration, which may introduce sampling bias. In addition, fixed 30-second arterial scanning delay without bolus tracking led to unstable arterial-phase contrast enhancement in our elderly cohort, explaining the lack of inter-group differences in arterial spectral parameters.

Clinically, LMR is a cheap, radiation-free peripheral marker with comparable grading efficacy, whereas GSI-CT requires extra radiation exposure and post-processing workloads without proven added diagnostic value. While dual-index assessment theoretically reflects both tumor perfusion and host immune status, the statistical evidence from this cohort does not support routine combination for preoperative grading. Subgroup analyses restricted to NMIBC and TURBT-only patients replicated the same non-significant comparison result between the combined model and LMR, confirming the robustness of our core conclusion. Tumor diameter acted as an independent confounder, yet λHU and LMR remained predictive after covariate adjustment.

### 4.2 Relationship between inflammatory markers and pathological features of bladder cancer

Systemic inflammation plays an important role in tumor progression, and markers including NLR, CAR, LMR, and PLR have been widely investigated in bladder cancer, particularly in relation to prognosis (29).

In the present study, LMR differed significantly between low- and high-grade tumors, suggesting a potential association with pathological grade. ROC analysis demonstrated moderate discriminatory ability (AUC = 0.835), characterized by high sensitivity but limited specificity. When combined with GSI-CT-derived quantitative parameters, the overall diagnostic performance was improved, indicating that integrating imaging biomarkers with systemic inflammatory markers may better reflect tumor biological behavior.

A possible biological explanation for this finding is that LMR reflects the balance between host antitumor immune response and protumor inflammatory activity (30). Lymphocytes play a key role in immune surveillance and tumor suppression, whereas monocytes may differentiate into tumor-associated macrophages, which promote tumor invasion, angiogenesis, and immune evasion (31). Therefore, a lower LMR may indicate both impaired antitumor immunity and enhanced protumor inflammation, which are features more commonly associated with higher-grade tumors (32).Of particular note, LMR achieved the highest single-marker AUC in our overall cohort, yet this peripheral inflammatory marker is highly non-specific and susceptible to severe confounding bias induced by active urinary tract infection, gross hematuria, chronic systemic inflammatory disorders, and recent steroid administration. A critical methodological limitation of this retrospective work is that no formal, predefined prospective exclusion criteria for these inflammatory interfering conditions were established at the study design and enrollment stage, allowing confounder-affected patients to enter the full primary cohort and introducing measurable noise to LMR quantification. To isolate the intrinsic grading performance of LMR without inflammatory disturbance, we performed a *post-hoc* retrospective sensitivity analysis by screening and removing 11 confounder-positive patients to form an inflammation-free restricted subgroup (n = 50). This inflammation-unconfounded subgroup represents a key supplementary exploratory analysis of our study, as it eliminates systemic inflammatory interference and reflects the biomarker’s true Tumor-related discriminatory capacity. In this restricted cohort, LMR’s AUC slightly declined to 0.812, which directly verifies that inflammatory comorbidities weaken its grading efficacy. However, given the lack of *a priori* exclusion rules, this subgroup analysis remains *post-hoc* and hypothesis-generating rather than a pre-planned primary endpoint, and its conclusions require large prospective external validation. Therefore, LMR cannot be adopted as an independent preoperative grading marker and should only be interpreted as an auxiliary index combined with lesion-specific spectral CT parameters.

In contrast, no significant associations were observed between tumor grade and other inflammatory indices, including NLR, CAR, and PLR. Several factors may account for these findings. First, the relatively small sample size may have limited statistical power. Second, systemic inflammatory markers are inherently non-specific and may be influenced by inter-patient variability, concurrent inflammatory or infectious conditions, and medication use. Importantly, strict exclusion criteria for concurrent inflammatory conditions, such as elevated white blood cell count, increased CRP levels, or urinary tract infection, were not applied in this study, which may have introduced potential confounding effects. Therefore, the results for NLR, CAR, and PLR should be interpreted with caution, and their role in histologic grading remains inconclusive. These markers may be more appropriately considered as complementary indicators rather than standalone predictors for tumor grading. The sensitivity analysis excluding patients who underwent radical cystectomy yielded findings comparable to those of the full cohort, suggesting that inclusion of this clinically selected subgroup did not materially influence the overall conclusions.

### 4.3 Clinical implication

The λHU + LMR combination may provide auxiliary preoperative risk hints for bladder cancer aggressiveness, but all related clinical inferences remain preliminary hypotheses pending large prospective multi-center validation. If future adequately powered trials confirm the model’s generalizability, clinicians may cautiously adjust surgical margins and surveillance frequency as a supplementary reference only. However, given our small single-center sample, lack of external validation and non-significant AUC gain, no adjustments to established EAU guideline-based treatment and surveillance protocols can be recommended based solely on this study’s data. Final clinical decisions still rely on postoperative histopathology, and the predictive value of this dual-marker panel needs further rigorous verification.

### 4.4 Limitations

This study has several key methodological and generalizability limitations:

First, Single-center retrospective design with limited sample size (n = 61) and no external validation cohort. No pre-study power analysis was performed to calculate the sample size required to detect small AUC differences between LMR and the combined λHU + LMR model; all subgroup findings remain exploratory and unconfirmed. All imaging was acquired on a single GE Discovery CT 750 HD scanner, restricting the external validity of our diagnostic thresholds and quantitative cut-offs to other manufacturers’ dual-energy CT platforms. Multi-center, multi-vendor cohorts with pre-specified power calculations are required to establish universal spectral CT grading benchmarks.

Second, Critical collinearity between pathological grade and muscle invasion status. Within our cohort, nearly all MIBC patients harboured high-grade urothelial carcinoma, leading to extreme correlation between Tumor aggressiveness and wall invasion. Severe multicollinearity prevented simultaneous inclusion of grade and stage within the same multivariate regression model, making it impossible to isolate their independent predictive effects on preoperative grading. Larger balanced cohorts containing both low-grade and high-grade MIBC cases are needed to disentangle these two clinical variables.

Third, No 3D spatial co-registration between CT Tumor ROI and surgical pathological specimens. All lesion ROIs were manually delineated on 2D axial CT slices without rigid 3D alignment to post-TURBT pathological resection maps. There exists inherent sampling bias: the CT-measured Tumor region may not precisely correspond to the exact tissue sections subjected to histopathological grading, potentially introducing measurement noise for all spectral quantitative indices.

Fourth, Methodological flaw related to LMR inflammatory confounders: No predefined prospective exclusion criteria were established for patients with active urinary tract infection, gross hematuria, chronic inflammatory disease or recent steroid use. The inflammation-free n = 50 subgroup was only generated via *post-hoc* retrospective medical record screening rather than pre-specified stratification at study design.

Fifth, Fixed 30-second arterial delay without automated bolus tracking in our elderly cohort (mean age 70.03). Variable cardiac output and systemic circulation times frequently missed peak arterial Tumor enhancement, which explains the non-significant arterial-phase spectral parameter differences observed in this work. Future protocols should implement bolus-triggered scanning.

Sixth, strong multicollinearity among venous IC, λHU and Zeff (VIF > 5). We retained only λHU in the final combined model to avoid unstable regression coefficients, which may discard minor independent predictive signals carried by IC and Zeff.

Seventh, Tumor multifocality data were collected but could not be adjusted in the current limited cohort due to highly unbalanced multifocal high/low-grade cases, which may introduce minor confounding bias.

Finally, only pure urothelial carcinoma patients were enrolled; rare variant histologies (squamous cell carcinoma, adenocarcinoma) and recurrent bladder Tumors were excluded, narrowing the population scope of our conclusions.

### 4.5 Future directions

Several critical research gaps remain unaddressed in this retrospective single-center analysis:

Large-scale, prospective multi-center cohorts are required for external validation of our predictive model. Future trials should adopt multiple dual-energy CT scanner vendors (not limited to GE platforms) to develop vendor-agnostic quantitative cut-offs with broader clinical generalizability.

To resolve the grade-stage collinearity limitation observed here, subsequent studies should prospectively recruit balanced numbers of low-grade and high-grade MIBC patients, enabling independent adjustment of Tumor stage and grade within multivariate regression frameworks to separate their respective predictive effects.

Standardized 3D spatial co-registration software should be integrated into imaging-pathology workflows to precisely match CT Tumor ROIs with histology resection specimens, eliminating sampling bias from unmatched 2D manual ROI selection.

Predefine strict prospective exclusion criteria for inflammatory confounders affecting LMR, and designate the inflammation-unconfounded subgroup as the primary analysis population in trial design.

Replace fixed 30s arterial delay with automated bolus tracking to reliably capture peak arterial Tumor enhancement in elderly patients with heterogeneous circulatory function.

Incorporate Tumor diameter as a covariate in regression models and expand enrolment to include variant histological types and recurrent bladder cancer patients for wider applicability analysis.

Integrate molecular Tumor biomarkers (TP53, FGFR3 mutation status) to clarify mechanistic links between spectral CT indices and Tumor aggressiveness.

Develop multimodal machine learning and artificial intelligence (AI predictive models by integrating spectral CT quantitative metrics, systemic inflammatory biomarkers and baseline clinical covariates. Multimodal AI frameworks can fuse lesion perfusion imaging features, peripheral immune indicators and clinical risk factors simultaneously to build more robust individualized preoperative grading and risk stratification tools for bladder cancer. As summarized by Katsimperis et al. in a recent review focusing on genitourinary oncology artificial intelligence applications, AI-assisted multimodal multimodal imaging analysis shows great potential to optimize preoperative risk stratification and tailor personalized surgical and surveillance regimens for urological malignancies (33). Further prospective multi-center data collection will support training, external validation and calibration of such combined machine-learning models to improve predictive stability and clinical translatability.

## 5 Conclusion

GSI-CT-derived quantitative parameters (IC, λHU, and Zeff) were significantly different between high-grade and low-grade bladder cancer, and combined evaluation with systemic inflammatory markers offers dual biological signals reflecting Tumor angiogenesis and host anti-Tumor immune status for exploratory preoperative grading. Crucially, venous λHU provided no statistically significant incremental diagnostic discrimination above standalone LMR in our complete cohort (AUC difference = 0.042, *P* = 0.061). No statistical evidence supports routine spectral CT use to improve bladder Tumor grading across general patient populations.

Preliminary *post-hoc* sensitivity analysis excluding patients with active inflammatory comorbidities tentatively suggested λHU may partially counteract LMR’s inflammatory-related performance reduction; however, this subgroup analysis was underpowered, retrospective and hypothesis-generating only, with no external validation to confirm consistency in larger cohorts.

Clinically, standalone LMR is an inexpensive, radiation-free blood marker with equivalent overall grading discrimination, while GSI-CT incurs extra imaging costs, ionising radiation and laborious quantitative analysis workloads. The unproven supplementary potential of spectral CT metrics cannot justify its routine addition to preoperative grading workflows based on the present single-center data.

The MIBC subgroup in our cohort suffered extreme imbalance of high/low-grade cases, preventing reliable assessment of model performance within this patient subset. All observations from this study are exploratory and cannot replace histopathology or MRI as standard staging/grading reference tools. The primary clinical interpretive scope of our findings is restricted to NMIBC patients. Any definitive evaluation of whether λHU carries meaningful additive grading utility requires large, prospectively designed, power-calculated multi-center cohorts for external validation.

## Data Availability

The original contributions presented in the study are included in the article/**Supplementary Material**. Further inquiries can be directed to the corresponding authors.

## Supplementary material

Supplementary Table 1 Multivariate logistic regression odds ratios and diagnostic AUC of the λHU + LMR model in the full cohort and restricted TURBT-only cohort excluding patients receiving radical cystectomy.

Supplementary Table 2 Multivariate logistic regression and ROC results with/without Tumor diameter adjustment (TURBT-only n=52).
