# Supplementary material for: Spectral CT-derived quantitative imaging parameters combined with inflammatory markers for exploratory preoperative grading of bladder cancer
Source: Front Oncol. 2026 Aug 12;16:1892226. doi: 10.3389/fonc.2026.1892226 (PMC13506437; doi:10.3389/fonc.2026.1892226)
Supplement: Supplementary file 1 [file Table1.docx]

Supplementary Table S1. Multivariate logistic regression odds ratios and diagnostic AUC of the λHU + LMR model in the full cohort and restricted TURBT-only cohort excluding patients receiving radical cystectomy

| Variable | Full cohort (n = 61) | Excluding RC (n = 52) |
| --- | --- | --- |
| λHU OR | 2.45 (95% CI 0.91–6.57) | 3.27 (95% CI 1.42–7.51) |
| LMR OR | 0.17 (95% CI 0.06–0.48) | 0.41 (95% CI 0.25–0.67) |
| Combined AUC | 0.877 | 0.872 |
| 95% CI | 0.787–0.955 | 0.779–0.951 |
