# Supplementary material for: Spectral CT-derived quantitative imaging parameters combined with inflammatory markers for exploratory preoperative grading of bladder cancer
Source: Front Oncol. 2026 Aug 12;16:1892226. doi: 10.3389/fonc.2026.1892226 (PMC13506437; doi:10.3389/fonc.2026.1892226)
Supplement: Supplementary file 2 [file Table2.docx]

Supplementary Table S2. Multivariate logistic regression and ROC results with/without Tumor diameter adjustment (TURBT-only n=52)

| Model | Variable | OR | 95% CI | *P* | Combined Model AUC (95% CI) |
| --- | --- | --- | --- | --- | --- |
| Unadjusted  (λHU + LMR) | λHU | 3.27 | 1.42–7.51 | 0.005 | 0.872 (0.779–0.951) |
|  | LMR | 0.41 | 0.25–0.67 | ＜0.001 |  |
| Adjusted  (λHU + LMR + Tumor diameter) | λHU | 2.98 | 1.31–6.78 | 0.009 | 0.892 (0.801–0.964) |
|  | LMR | 0.45 | 0.28–0.72 | 0.001 |  |
|  | Tumor diameter | 1.72 | 1.12–2.63 | 0.013 |  |
